# In Memoriam—Dr. John L. Ferson

**Published:** 1896-07

**Authors:** 


### IN MEMORIAM—DR. JOHN L. FERSON.

The committee appointed to draft a set of resolutions in memory of Dr. John L. Ferson, deceased, beg to report the following:

WHEREAS, In the dispensation of an all-wise Providence, our esteemed friend and fellow-member, John L. Ferson, M. D., has been removed from the scene of his labors and good works in this world, and has entered upon his rest in the other and better world; and,

WHEREAS, Many years of professional fellowship and acquaintance have endeared the deceased to each of us, and have demonstrated in him superior acquirements as a physician and noble qualities as a man; and,

WHEREAS, By his death this Society has been deprived of one of its most loyal and earnest members, and his professional associates of the benefit of his judicious counsel and wise advice; therefore,

*Resolved*, That in the death of Dr. Ferson, the Homœopathic Medical Society of Allegheny County has lost a faithful and valued member, and the system of medicine which he so steadfastly and ably supported has lost one of its truest and most consistent advocates.

*Resolved*, That we tender to the bereaved family of the deceased our heartfelt sympathy and condolence, and fervently hope that their affliction will be less keenly felt by the assurance that he will be gratefully remembered by a community in which he was so highly esteemed and universally beloved.

*Resolved*, That a copy of these resolutions be spread upon the minutes of the Society, be sent to the family of our deceased fellow-member, and to the principal medical journals of our school.

*Resolved*, That as the last act of respect and esteem that it will be our privilege to pay Dr. Ferson, the Society attend in a body the funeral services, Thursday morning, July 9th, at his late residence, Wylie Avenue.

(Signed)

W. J. Martin, M. D.
J. B. McClelland, M. D.
J. F. Cooper, M. D.
J. C. Burgher, M. D.
T. A. Willard, M. D.

